# Using unilateral stimulation to create a reference for bilateral fusion judgments

**DOI:** 10.1121/10.0007058

**Published:** 2021-11-02

**Authors:** Grace Hyerin Kim, Justin M. Aronoff

**Affiliations:** Speech and Hearing Science Department, University of Illinois at Urbana-Champaign, 901 S. 6thStreet, Champaign, Illinois 61801, USA hyerinkim2023@u.northwestern.edu, jaronoff@illinois.edu

## Abstract

Measuring binaural fusion can be challenging, especially with bilateral cochlear implant (CI) users. This study validated a technique of using unilateral stimulation to create a reference for measuring fusion. Seven bilateral CI users listened to stimuli randomly presented to the right, left, or both ears. Participants indicated the size, number, and location of the resulting image(s) they perceived. The participants had largely unitary, punctate percepts that were lateralized to the stimulated ear for unilateral stimuli. The image was centered but more diffuse when the stimuli were presented bilaterally. The results suggest unilateral stimuli can provide a reference for binaural fusion.

## 1. Introduction

Having access to binaural cues through bilateral cochlear implants (CI) improves speech understanding in noise and localization (Dunn *et al.*, 2010; van Hoesel, 2004; Litovsky *et al.*, 2012). However, access to binaural cues may rely on binaurally integrating signals from the two ears, as suggested by the correlation between binaural fusion and localization (Suneel *et al.*, 2017). Measuring binaural fusion, defined as the percept of a unitary, punctate sound when a signal is presented to both ears (Kan *et al.*, 2013; Suneel *et al.*, 2017), can be difficult with both normal hearing (NH) participants and bilateral CI users. Measuring binaural fusion with bilateral CI users can be particularly challenging because, in addition to the general challenge of explaining what is meant by “fused” to naive participants, some bilateral CI users do not perceive any sounds as fully fused (Fitzgerald *et al.*, 2015; Kan *et al.*, 2013). This makes it difficult to provide an example to this group of a fused sound. This means that bilateral CI users often lack a reference for making fusion judgments.

Techniques can be used to help indicate what is meant by “fused,” such as having participants indicate fusion by marking the spatial extent, location, and number of images that are perceived in the head for a given signal (Kan *et al.*, 2013). However, such methods still rely on an internal reference of what is meant by different sized images in the head. This makes judgments susceptible to the particular set of stimuli used in a given experiment (Poulton, 1979), reducing the generalizability of the results. This suggests that there is a need for a reference that can be used across experiments.

One possible method for providing CI users with a reference for making binaural fusion judgments is to use unilateral stimulation, which, in theory, should be perceived as unitary and punctate, a similar perception as a fused stimulus. The goal of this study is to determine if unilateral stimulation provides a more unitary, punctate percept than bilateral stimulation for bilateral CI users. This would allow unilateral stimulation to be used as a reference of a unitary, punctate percept.

## 2. Methods

### 2.1 Participants

Seven bilateral CI users participated in this experiment. All participants used Cochlear brand devices. Participant demographics are shown in Table 1.

**Table 1.** Participant demographics.

Subject	Gender	Age	Onset of Hearing Loss (y)		Duration of CI use (y)	
			Left	Right	Left	Right
I12	Female	48	Birth	Birth	9	7
I37	Male	67	54	54	7	5
I38	Male	76	49	42	8	8
I42	Female	65	Childhood hearing loss	Childhood hearing loss	6	24
I59	Male	51	30	30	2	3
I63	Female	35	5	5	10	11
I68	Female	18	Birth	Birth	8	16

### 2.2 Stimuli

There were a total of eight stimuli, each consisting of a vocalization of /a/ collected in a previous study (Kirchner *et al.*, 2020). Multiple vocalizations, recorded from different speakers, were used in a random order to help participants maintain attention. A sustained vowel was selected because it is an ecologically valid, broadband stimulus. Vocalizations were recorded at a sampling rate of 44.1 kHz. Each vocalization was produced by a different individual and sustained for approximately 5 s. One second clips, selected to minimize the F0 variability, were excised from each recording and used as the stimuli. The fundamental frequencies ranged from 81–253 Hz.

### 2.3 Procedures

Stimuli were presented to the participants via the auxiliary input port of laboratory speech processors using a modified Edirol (Hamamatsu, Shizuoka, Japan) UA-1EX sound card with built in electrical isolation. Prior to starting the test, participants were presented with the bilateral stimuli and asked if the sound was centered in their head and at a comfortable listening level. If it was not, participants were asked to change the volume on one or both processors until the image was centered and still at a comfortable listening level.

**Fig. 1.**
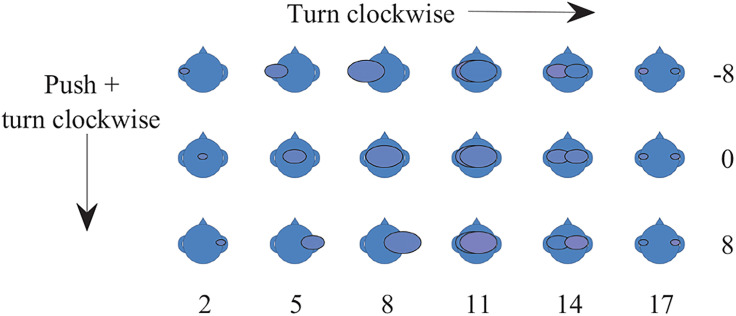
Examples of images participants saw as they moved the dial. The numbers reflect the scores that correspond to each image. Scores along the bottom indicate the number of steps along the fusion scale and scores along the side indicate the number of steps along the lateralization scale left (negative values) or right (positive values) of center.

For the test, participants were asked to indicate the location and size of the image in their head resulting from the stimuli, using a procedure similar to that used in Suneel *et al.* (2017). Participants indicated their percept using a dial (Powermate, Griffin Technology, Nashville, TN). By turning the dial, they could decrease or increase the size of the image to report a punctate or diffuse sound, respectively (see Fig. 1). Continuing to turn the dial clockwise would result in two separate sound images. Additionally, participants could indicate the lateralization of the sound by pushing down and turning the dial. When they indicated that there was a single, unitary percept, pushing down and turning the dial moved the image to the left or right. When they indicated there were two separate sounds, pushing down and turning the dial allowed them to indicate which image was more dominant and how dominant it was (see Fig. 1). The dial created discrete steps when turned, with each indicated by a change in the image on the screen. A total of 18 steps were possible for the fusion measure and 21 steps for the lateralization measure. The data consisted of two scores. One indicated the number of steps the dial was turned. The other indicated the number of steps the dial was turned while pushed down (see Fig. 1). Prior to the experiment, the participant was provided instruction as to how to use the dial and practiced manipulating the images using the dial.

Participants could repeat each stimulus as many times as they wished. There was a total of 72 stimuli (8 recordings × 3 listening conditions × 3 trials per recording/listening condition combination). Trials were divided across three blocks, with an equal likelihood of the stimuli being delivered to either the right ear, the left ear, or both ears in each block. The participants were not told that the stimulus would sometimes be delivered to only one ear.

## 3. Results

Robust statistical techniques were used to minimize the effects of outliers and non-normality in the data (see the Appendix in Aronoff *et al.*, 2016). The number of discrete steps used when turning the dial was used as the fusion score. The number of discrete steps used when pushing down and turning the dial was used as the lateralization score. The median fusion score was calculated for each participant. In addition to the fusion scores for the bilateral condition, scores for the left and right ear conditions were combined into a unilateral fusion score by taking the median score for all trials delivered unilaterally, regardless of the ear to which they were delivered. The median lateralization score was calculated for each participant and condition, separately analyzing the left and right ear condition.

### 3.1 Fusion

**Fig. 2.**
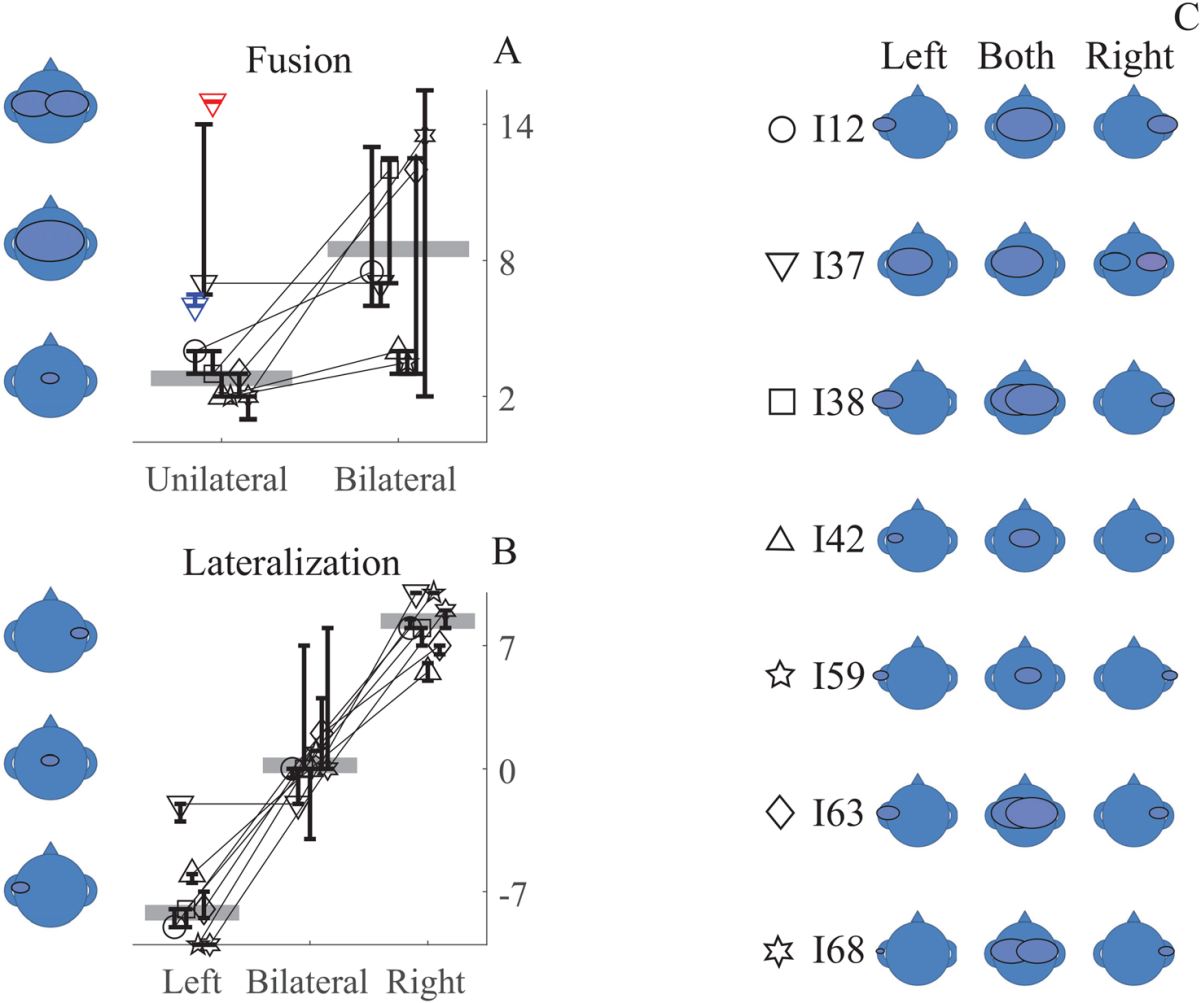
(A) Median fusion scores per participant and 95% confidence interval of the median. Unilateral scores are pooled across left and right ear conditions. I37's unilateral data are also shown for their left (blue triangle) and right (red triangle) CI. The horizontal gray bars indicate the group median. See panel C for the subject numbers corresponding to each symbol. (B) Median lateralization scores per participant and 95% confidence interval of the median. The horizontal gray bars indicate the group median. See panel C for the subject numbers corresponding to each symbol. (C) Depiction of the image that corresponds to the median fusion and lateralization score for each participant and condition.

Individual scores are provided in Figs. 2(a) and 2(c). Scores for the unilateral and bilateral conditions were compared using the Wilcoxon Signed Rank test. This indicated that there was a significant difference between the two conditions (*p* = 0.04), with greater fusion/more punctate percepts (i.e., lower scores) for the unilateral condition. Additionally, there was notably more variability in each individual's responses in the bilateral condition.

### 3.2 Lateralization

Individual scores are provided in Figs. 2(b) and 2(c). Scores were compared across conditions using the Friedman test. There was a significant effect of condition [Χ^2^_F_(2) = 13.6; *p* < 0.01]. Pairwise comparisons using Wilcoxon Signed Rank test indicated that all conditions significantly differed from each other (all *p* values < 0.05). Additionally, as with the fusion data, there was notably more variability in each individual's responses in the bilateral condition.

## 4. Discussion

Unilateral stimulation resulted in a single, punctate percept. The percept was lateralized to the side of stimulation, a common, but not universal result for bilateral cochlear implant users (Gordon *et al.*, 2014; Litovsky *et al.*, 2009). The individual participant results were largely consistent with the group results, although one participant (I37) indicated a non-singular percept for one unilateral condition. Participants were consistent in their responses to the unilateral stimuli, as indicated by the small 95% confidence interval. Even I37, who had a large 95% confidence interval when pooling across ears, was consistent in his response for each ear, with the large confidence interval when pooling across ears reflecting the different response when using the left versus the right ear.

The results confirmed our hypothesis that bilateral stimulation would result in a less unitary and/or punctate percept than unilateral stimulation. In the absence of an objective measure of fusion, it is not possible to independently validate the bilateral condition fusion score. However, the pattern of results is consistent with findings in the literature that binaural fusion is poor for some bilateral CI users (Fitzgerald *et al.*, 2015) and with CI simulations with broadband sounds (Aronoff *et al.*, 2015; Suneel *et al.*, 2017). Future studies should directly compare bilateral fusion scores with and without a unilateral reference to determine if the unilateral condition significantly changes response patterns and helps the participant perform the subjective task.

There was a notable increase in the variability of the responses for the bilateral condition. This does not appear to simply reflect increased difficulty with fusion judgments, since this increased variability was also seen with the lateralization data. Instead, it suggests that participants had a more variable percept when using the two ears together. This more variable percept may have occurred because lateralization of a sound is much more sensitive to small differences across stimuli for sounds near the midline, such as those in the bilateral condition, than for lateralized sounds, such as those in the unilateral conditions (Yost, 1981).

An important caveat is that the use of a modulated signal (i.e., presenting a sustained vowel through a processor generally resulted in an electrical signal that was modulated at a rate equivalent to the F0 of the voice) may have increased fusion scores in the bilateral condition since modulations can improve fusion (Oh and Reiss, 2018). This potentially minimized the difference between unilateral and bilateral fusion scores. As a result, the study may have underestimated the detrimental effects of bilateral stimulation on binaural fusion.

A second caveat is that the sample size for this study was small, and future studies with larger populations are needed. This would be particularly important for determining the prevalence of non-unitary percepts for unilateral stimuli, such as was seen with I37. I37's results suggest that, for some participants, a unilateral stimulus will be perceived as a diffuse or unfused sound. This is consistent with previous studies showing that, participants, particularly those with asymmetric hearing loss, can show a wide range of perceived lateralization with unilateral stimuli (Cadieux *et al.*, 2013; Kumpik and King, 2019). Unclear lateralization may lead to the percept of an unfused sound. It is possible to use the unilateral data to determine if unilateral stimuli result in a unitary, punctate percept on a case-by-case basis. However, understanding the prevalence of such non-unitary percepts is critical to determine the extent to which the use of a unilateral stimulus as a reference can be beneficial for measuring binaural fusion in the larger CI population.

In summary, the results indicate that unilateral stimuli can provide CI users with a reference of a unitary, punctate auditory image. This could potentially increase the accuracy of fusion measurements with CI users as well as with other populations.
